# Reply to: Factors associated with mortality in mechanically ventilated patients with severe acute respiratory syndrome due to COVID-19 evolution

**DOI:** 10.62675/2965-2774.20240213-en

**Published:** 2024-10-01

**Authors:** Luis Felipe da Fonseca Reis, João Paulo Arruda de Oliveira, Arthur de Sá Ferreira, Agnaldo José Lopes

**Affiliations:** 1 Centro Universitário Augusto Motta Postgraduate Program in Rehabilitation Sciences Rio de Janeiro RJ Brazil Postgraduate Program in Rehabilitation Sciences, Centro Universitário Augusto Motta - Rio de Janeiro (RJ), Brazil.

To the Editor

We thank Dr. Arroyo-Sanchez and R. Aguirre-Mejía^(1)^ for their interest and praise regarding our study entitled "Factors associated with mortality in mechanically ventilated patients with severe acute respiratory syndrome due to COVID-19 evolution".^(2)^

In their letter to the editor, the authors state that data on prone mechanical ventilation were not used in the analysis model.^(1)^ Prone mechanical ventilation is a recommended approach in patients with moderate and severe acute respiratory distress syndrome (ARDS). However, methodologically, predictive models must be built on the basis of the characteristics of the population rather than on the basis of the proposed therapeutic interventions.^(3)^ Our predictive model was built to answer, in a pandemic scenario of uncertainty, which clinical and anthropometric characteristics could explain the prognosis of patients with severe acute respiratory syndrome due to the evolution of coronavirus disease 2019 (COVID-19). All the most important prognostic scores for individual mortality in the intensive care unit, such as the Acute Physiology and Chronic Health Evaluation (APACHE) III, Simplified Acute Physiology Score (SAPS) III and Sequential Organ Failure Assessment (SOFA), were constructed considering the characteristics of the individuals, and they have moderate to high predictive accuracy of mortality. A cohort involving more than 280,000 patients demonstrated that individual characteristics represent the main prognostic factor in ARDS due to COVID-19.^(4)^

Another point, highlighted by the authors of extreme relevance to the debate, is the paradox of obesity, which has previously been described as having a protective effect or at least as not being a factor of worse prognosis in patients with ARDS. However, other studies have differing results and demonstrate that a body mass index (BMI) > 40kg/m^2^ is the strongest predictor of hospital mortality (HR: 3.147, 95% confidence interval [95%CI] 1.00 - 9.89), making it an independent risk factor for hospital mortality.^(5)^ Other studies present similar results, where BMI was significantly associated with in-hospital mortality (HR = 2.49 per 10 BMI units; 95%CI 1.69 to 3.70; p < 0.001) even after adjustment for sex, age and obesity-related comorbidities (adjusted HR = 3.50; 95%CI from 2.03 to 6.02).^(6)^ In our study, a higher BMI (≥ 30kg/m2) was associated with a 17% increase in the risk of mortality (RR: 1.17; 95%CI 1.11 - 1.20; p < 0.001), and our hypothesis is supported because patients with obesity present activation of the inflammatory cascade with a greater concentration of proinflammatory cytokines produced by adipose tissue, compromising the immune response, in addition to being associated with hypercoagulability disorders, which are associated with a worse prognosis during the evolution of COVID-19.^(5,6)^ Patients with COVID-19 have marked increases in the levels of IL-1β, IFN-γ, and monocyte chemoattractant protein-1 (MCP-1).^(4-6)^ On the other hand, obesity is considered a chronic inflammatory condition characterized by the upregulation of the interleukins TNF-, IL-1, IL-6 and MCP-1, which cause insulin resistance, as well as cardiovascular and respiratory problems.^(7)^ Thus, although the obesity paradox is described as a possible protective effect in ARDS, in ARDS, owing to the evolution of COVID-19, this assumption is not consensually supported, as demonstrated in our multicenter retrospective cohort. Furthermore, the authors recognize that our results must be analyzed as data extracted from a historical cohort and analyzed in accordance with the limitations of longitudinal studies.
