# Supplementary material for: Reply to: Factors associated with mortality in mechanically ventilated patients with severe acute respiratory syndrome due to COVID-19 evolution
Source: Crit Care Sci. 2024 Oct 1;36:e20240213en. doi: 10.62675/2965-2774.20240213-en (PMC11463990; doi:10.62675/2965-2774.20240213-en)
Supplement: Translation

# Translation: Resposta para: Fatores associados à mortalidade em pacientes ventilados mecanicamente com síndrome respiratória aguda grave por evolução da COVID-19

**DOI:** 10.62675/2965-2774.20240213-pt

**Authors:** Luis Felipe da Fonseca Reis, João Paulo Arruda de Oliveira, Arthur de Sá Ferreira, Agnaldo José Lopes

**Affiliations:** 1 Rio de Janeiro RJ Brasil Programa de Pós-Graduação em Ciências da Reabilitação, Centro Universitário Augusto Motta - Rio de Janeiro (RJ), Brasil.

Ao Editor

Agradecemos ao Dr. Arroyo-Sanchez e a R. Aguirre-Mejía^(1)^ por seu interesse e elogios em relação ao nosso estudo intitulado *Factors Associated with Mortality in Mechanically Ventilated Patients with Severe Acute Respiratory Syndrome due to COVID-19 Evolution*.^(2)^

Em sua carta ao editor, os autores afirmam que não foram usados dados da ventilação mecânica em decúbito ventral no modelo de análise.^(1)^ A ventilação mecânica em decúbito ventral é uma abordagem recomendada em pacientes com síndrome do desconforto respiratório agudo (SDRA) moderado e grave. No entanto, metodologicamente, os modelos preditivos devem ser construídos com base nas características da população, não com base nas intervenções terapêuticas propostas.^(3)^ Nosso modelo preditivo foi construído para responder, em um cenário pandêmico de incerteza, quais características clínicas e antropométricas poderiam explicar o prognóstico de pacientes com síndrome respiratória aguda grave devido à evolução da doença do coronavírus 2019 (COVID-19). Todos os escores prognósticos mais importantes para a mortalidade individual na unidade de terapia intensiva, como o *Acute Physiology and Chronic Health Evaluation* (APACHE) III, o *Simplified Acute Physiology Score* (SAPS) III e o *Sequential Organ Failure Assessment* (SOFA), foram construídos considerando as características dos indivíduos e têm precisão preditiva de mortalidade moderada a alta. Uma coorte envolvendo mais de 280 mil pacientes demonstrou que as características individuais representam o principal fator prognóstico na SDRA devido à COVID-19.^(4)^

Outro ponto destacado pelos autores e de extrema relevância para o debate é o paradoxo da obesidade, que já foi descrita como tendo um efeito protetor ou, pelo menos, como não sendo um fator de prognóstico desfavorável em pacientes com SDRA. No entanto, outros estudos apresentam resultados diferentes e demonstram que índice de massa corporal (IMC) > 40 kg/m^2^ é o mais forte preditor de mortalidade hospitalar (hazard ratio [HR] de 3,147, intervalo de confiança de 95% [IC95%] 1,00 - 9,89), o que o torna um fator de risco independente para mortalidade hospitalar.^(5)^ Outros estudos apresentam resultados semelhantes, em que o IMC foi significativamente associado à mortalidade intra-hospitalar (HR de 2,49 por 10 unidades de IMC; IC95% 1,69 - 3,70; p < 0,001), mesmo após o ajuste para sexo, idade e comorbidades relacionadas à obesidade (HR ajustado de 3,50; IC95% 2,03 - 6,02).^(6)^ Em nosso estudo, IMC mais elevado (≥ 30 kg/m^2^) foi associado a aumento de 17% no risco de mortalidade (razão de risco [RR] 1,17; IC95% 1,11 - 1,20; p < 0,001), e nossa hipótese é apoiada porque os pacientes com obesidade apresentam ativação da cascata inflamatória com maior concentração de citocinas pró-inflamatórias produzidas pelo tecido adiposo, comprometendo a resposta imunológica, além de estarem associados a distúrbios de hipercoagulabilidade, que estão associados a um prognóstico desfavorável durante a evolução da COVID-19.^(5,6)^ Pacientes com COVID-19 apresentam aumentos acentuados nos níveis de interleucina 1 beta (IL-1β), interferon gama (IFN-γ) e proteína quimioatraente de monócitos-1 (MCP-1).^(4-6)^ Por outro lado, a obesidade é considerada uma condição inflamatória crônica caracterizada pela regulação positiva das interleucinas fator de necrose tumoral (TNF-), IL-1, IL-6 e MCP-1, que causam resistência à insulina, bem como problemas cardiovasculares e respiratórios.^(7)^ Assim, embora o paradoxo da obesidade seja descrito como um possível efeito protetor na SDRA, devido à evolução da COVID-19, essa suposição não é consensualmente apoiada, conforme demonstrado em nossa coorte retrospectiva multicêntrica. Além disso, os autores reconhecem que nossos resultados devem ser analisados como dados extraídos de uma coorte histórica e de acordo com as limitações dos estudos longitudinais.
